# Multidisciplinary Evidences that *Synechocystis* PCC6803 Exopolysaccharides Operate in Cell Sedimentation and Protection against Salt and Metal Stresses

**DOI:** 10.1371/journal.pone.0055564

**Published:** 2013-02-06

**Authors:** Thichakorn Jittawuttipoka, Mariane Planchon, Olivier Spalla, Karim Benzerara, François Guyot, Corinne Cassier-Chauvat, Franck Chauvat

**Affiliations:** 1 UMR8221, CEA, CNRS, Université Paris-Sud, iBiTec-S, LBBC, Bat 142 CEA-Saclay, F-91191 Gif sur Yvette CEDEX, France; 2 CEA, DSM/IRAMIS/SIS2M/LIONS, UMR CEA-CNRS 3299, F-91191 Gif sur Yvette, France; 3 UMR 7590, CNRS, Universités Paris 6 et 7, IPGP, Institut de Minéralogie et de Physique des Milieux Condensés, 4, place Jussieu, 75252 Paris Cedex 05, France; University of Groningen, Groningen Institute for Biomolecular Sciences and Biotechnology, The Netherlands

## Abstract

Little is known about the production of exopolysaccharides (EPS) in cyanobacteria, and there are no genetic and physiological evidences that EPS are involved in cell protection against the frequently encountered environmental stresses caused by salt and metals. We studied four presumptive EPS production genes, *sll0923, sll1581, slr1875* and *sll5052*, in the model cyanobacterium *Synechocystis* PCC6803, which produces copious amounts of EPS attached to cells (CPS) and released in the culture medium (RPS) as shown here. We show that *sll0923, sll1581, slr1875* and *sll5052* are all dispensable to the growth of all corresponding single and double deletion mutants in absence of stress. Furthermore, we report that *sll0923*, *sll1581* and *slr1875* unambiguously operate in the production of both CPS and RPS. Both *sll1581* and *slr1875* are more important than *sll0923* for CPS production, whereas the contrary is true for RPS production. We show that the most EPS-depleted mutant, doubly deleted for *sll1581* and *slr1875*, lacks the EPS mantle that surrounds WT cells and sorbs iron in their vicinity. Using this mutant, we demonstrate for the first time that cyanobacterial EPS directly operate in cell protection against NaCl, CoCl_2_, CdSO_4_ and Fe-starvation. We believe that our EPS-depleted mutants will be useful tools to investigate the role of EPS in cell-to-cell aggregation, biofilm formation, biomineralization and tolerance to environmental stresses. We also suggest using the fast sedimenting mutants as biotechnological cell factories to facilitate the otherwise expensive harvest of the producer cell biomass and/or its separation from products excreted in the growth media.

## Introduction

A wide-range of bacteria synthesize and secrete extracellular polymeric substances, mainly of polysaccharidic nature (exopolysaccharides (EPS)), which are involved in the tolerance to environmental stresses [1]. In addition, EPS have valuable biotechnological applications [2], and are involved in the formation and maintenance of biofilms, which have a high relevance to human health [3], water treatment and agriculture [4]. Two types of EPS can be distinguished: (i) capsular EPS (CPS), which are attached to the cell surface and (ii) EPS that are released into the surrounding environment (RPS). The synthesis of EPS comprises typically four distinct steps occurring in various compartments 1) the activation of monosaccharides and conversion into sugar nucleotides into the cytoplasm; 2) the assembly of the repeat units by sequential addition of sugar on a lipid carrier by glycosyltransferase; 3) the polymerization of the repeat units at the periplasmic face of the plasma membrane; and 4) the export of the polymer to the cell surface.

In cyanobacteria, the important photosynthetic prokaryotes, which colonize most waters and soils of our planet and produce a large part of the oxygen [5] and biomass for the food chain [6], EPS are proposed to operate in the protection against environmental stresses [7]. Furthermore, cyanobacterial EPS are also regarded as being involved in the biomineralization of calcium (and/or magnesium) carbonates, sometimes leading to the formation of stromatolites [8]. Cyanobacterial EPS, which harbour six to twelve types of monosaccharides, are more complex than the EPS formed by other bacteria or eukaryotic microalgae, which usually contain less than four monosaccharides [1]. Hence, the complexity of cyanobacterial EPS gives rise to speculations on how many genes may be involved in their production. In addition, cyanobacterial EPS are usually strongly anionic since they contain one or two different uronic acids, as well as sulphate groups, a rare feature among bacteria. The presence of negatively charged EPS surrounding cyanobacterial cells may play an important role in the sequestration of metal cations. On one hand, this can create a microenvironment enriched in those metals that are essential for cell growth but occur in limited amounts in the environment. On the other hand, the presence of EPS around the cells can also prevent direct contact between the cells and toxic heavy metals such as cadmium (Cd) and cobalt (Co), which are intensively spread out in the environment by human industries [9]. Consequently, EPS-producing cyanobacteria are regarded as promising chelating agents for biosorption and removal of heavy metals from contaminated waters [7]. EPS have also been proposed to contribute to the protection of cyanobacteria against other environmental stresses such as dessication, UV-light, biomineralization, and salt stress. However, direct experimental evidence demonstrating the ecological roles attributed to cyanobacterial EPS are scarce [7]. To our knowledge only one paper reported the analysis of cyanobacterial genes involved in EPS production [10]. This study was carried out in the unicellular model strain *Synechocystis* PCC6803 (hereafter *Synechocystis*) that possesses a fully sequenced (http://genome.kazusa.or.jp/cyanobase/) and easily manipulable genome [11]–[14], which encodes complex EPS with 12 types of monosaccharides [1]. In their paper, Foster and co-workers showed that the five genes cluster sll1722-sll1726 encoding presumptive glycosyltransferases is involved in the production of EPS and the protection against the light and H_2_O_2_ stresses [10]. However, this previous study did not distinguish between CPS and RPS, and did not investigate the influence of EPS on the tolerance to salt and metal stresses, or the formation of biofilm.

In the present study, we have thoroughly analyzed four *Synechocystis* genes, *slr1875*, *sll1581*, *sll0923* and *sll5052*, which share some sequence homology with the EPS production genes *exoD (slr1875)*, *gumB (sll1581)* and *gumC* (*sll0923*, *sll5052)* from non-photosynthetic bacteria. We report that these four *Synechocystis* genes are all dispensable to cell growth under standard laboratory conditions (all single and double-deletion mutants grow as healthy as the WT strain). Genes *sll0923*, *sll1581* and *slr1875* play an important role in EPS production, unlike *sll5052*. EPS are abundant in the WT strain where they form a thick mantle that surrounds the cells and sorbs high amounts of iron atoms. Furthermore, we show that EPS play a crucial role in the tolerance to stresses triggered by NaCl, CdSO_4_, CoCl_2_, or Fe starvation. These findings are the first genetic and physiological evidences that EPS protect cyanobacteria against salt and metal stresses.

## Materials and Methods

### Bacterial Strains, Growth and Survival Analyses

*Synechocystis* PCC6803 was grown under continuous agitation (180 rpm) and white light (2,500 luxes; 31.25 µE m^−2^ s^−1^) at 30°C on mineral medium (MM) corresponding to BG11 medium [15] enriched with 3.78 mM Na_2_CO_3_ [16]. *E. coli* TOP10 (Invitrogen) used for gene manipulation was grown on LB at 37°C. Antibiotic selection was performed with kanamycin (Km) 50 µg ml^−1^, streptomycin (Sm) 2.5 µg ml^−1^ or spectinomycin (Sp) 2.5 µg ml^−1^ for *Synechocystis*; and ampicillin (Amp) 100 µg ml^−1^, Km 50 µg ml^−1^ or Sp 75 µg ml^−1^ for *E. coli*. For NaCl challenges, *Synechocystis* cultures grown three times up to mid-exponential phase (OD_580_ = 0.5 units, i.e. 2.5×10^7^ cells.ml^−1^) were inoculated (initial OD_580_ = 0.02) into fresh MM medium with or without NaCl 0.9 M, and OD_580_ was measured at time intervals. For survival analyses, 1 ml aliquots of mid-exponential phase cultures were incubated for 3 h with various concentrations of CoCl_2_ or CdSO_4_, washed with ultrapure water (UPW, Purelab, Elga), and spread on MM plates after appropriate dilutions with MM. Colonies generated by surviving cells were counted after for 5–7 days under standard conditions.

### Construction of the DNA Cassette for Targeted Deletion of the *sll0923*, *sll1581*, *slr1875*, and *sll5052* Genes

The *Synechocystis* DNA regions (about 300 bp in length) flanking the protein coding sequence (CS) of the studied genes were independently amplified by PCR, using specific oligonucleotides primers (Table S1). These two DNA regions were joined through standard PCR-driven overlap extension [17] in a single DNA segment harbouring a *Sma*I restriction site in place of the studied CS. After cloning in pGEMT (Promega) the resulting plasmids (Table 1) were opened at the unique *Sma*I where we cloned the Km^r^ cassette (a *Hinc*II fragment of pUC4K) or the Sm^r^/Sp^r^ cassette amplified by PCR and digested with *Eco*RV) in the same orientation as the CS it replaced (Table 1). The resulting deletion cassettes were verified by PCR and nucleotide sequencing (Big Dye kit, ABI Perking Elmer), before and after transformation [18] to *Synechocystis*.

**Table 1:** Characteristics of the plasmids used in this study.

Plasmids	Relevant features	Reference
pGEMT	Amp^r^ AT overhang cloning vector	Promega
pUC4K	Source of the Km^r^ marker gene	Pharmacia
pFC1	Source of the Sm^r^/Sp^r^ marker gene	[12]
pΔsll0923	pGEMT with the *sll0923* gene and its two 0.5 kb flanking sequences, where the coding sequence(CS; from 28 bp to 2192 bp) was replaced by a *Sma*I site	This study
pΔsll0923::Km^r^	pΔsll0923 with the Km^r^ marker inserted into its unique *Sma*I site	This study
pΔsll0923::Sm^r^/Sp^r^	pΔsll0923 with the Sm^r^/Sp^r^ marker inserted into its unique *Sma*I site	This study
pΔsll1581	pGEMT with *slr1581* gene and its two 0.5 kb flanking sequences, where the *sll1581* CS (from 43 bpto 1419 bp) was replaced by a *Sma*I site	This study
pΔslr1581::Km^r^	pΔsll1581 with the Km^r^ marker inserted into its unique *Sma*I site	This study
pΔslr1581::Sm^r^/Sp^r^	pΔslr1875 with the Sm^r^/Sp^r^ marker inserted into its unique *Sma*I site	This study
pΔslr1875	pGEMT with the *slr1875* gene and its two 0.3 kb flanking sequences, where the CS (from 16 bp to 568 bp)was replaced by a *Sma*I site	This study
pΔslr1875::Km^r^	pΔslr1875 with the Km^r^ marker inserted into its unique *Sma*I site	This study
pΔslr1875::Sm^r^/Sp^r^	pΔslr1875 with the Sm^r^/Sp^r^ marker inserted into its unique *Sma*I site	This study
pΔsll5052	pGEMT with the *slr1581* gene and its two 0.3 kb flanking sequences, where the *sll5052* CS (from 100 bpto 2202 bp) was replaced by a *Sma*I site	This study
pΔsll5052::Km^r^	pΔsll5052 with the Km^r^ marker inserted into its unique *Sma*I site	This study
pΔslr5052::Sm^r^/Sp^r^	pΔsll5052 with the Sm^r^/Sp^r^ marker inserted into its unique *Sma*I site	This study

CS: coding sequence.

### Extraction and Quantification of Exopolysaccharides (EPS), and ATR-FITR Spectroscopy

EPS were extracted mostly as described [19]. 30 ml of late log phase cultures (OD_580_ = 0.7) grown for three days under standard conditions were harvested by a 30 min centrifugation (5,000 rpm) at room temperature (RT°) to separate the supernatants containing the released EPS (RPS) from the cell pellets containing the capsular EPS (CPS) attached to the cells. RPS were recovered from filtered supernatants (0.45 µm, Pall) after overnight precipitation with 2 volumes of chilled ethanol at −20°C, centrifugation at 13,000 rpm for 30 min at 4°C, washing with 95% ethanol, drying under air, and re-suspension in a 1 ml UPW. CPS were recovered from cell pellets, washed, re-suspended in 1 ml of UPW, boiled for 15 min at 100°C and centrifuged at 13,000 rpm at RT° to eliminate cell debris. Total carbohydrate contents of RPS and CPS were measured by the phenol-sulfuric method, and the amount of EPS was calculated as the average ratio of the EPS quantity over total proteins quantified with Bradford assay, using BSA as a standard (Biorad protein assay). For ATR-FITR spectroscopy, cells were deposited on an ATR crystal of ZnSe in a FTIR Nicolet 870 spectrometer, equipped with a cadmium telluride (MCT) detector. A total of 50 scans at a resolution of 4 cm^−1^ was averaged for each studied strains, and the spectrum of the MM growth medium alone was subtracted.

### Cell Sedimentation and Electrophoretic Mobility Assays

*Synechocystis* mid-log phase cultures were either stored motionless on the bench for 18 days prior to photographs, or transferred into disposable folded capillary cells with gold covered electrodes (Malvern).

Electrophoretic mobility was measured at the 17° fixed scattering angle with a Zetasizer Nano ZS instrument (Malvern) equipped with a 633 nm laser, using runs performed at a voltage of 150 V and a frequency of 285 Hz. The values were automatically converted to Zeta potentials using the Smoluchowski’s equation.

### Scanning Electron Microscopy and Energy Dispersive X-ray Analyses

Cells from late log phase cultures (OD_580_ = 0.7) were fixed overnight with glutaraldehyde (2.5%) and alcian blue (0.15%), washed twice with UPW and dried in CO_2_ critical point dryer (BAL-TEC CPD030) as described [20]. Samples were mounted on aluminium stubs using double-sided carbon tape, coated with carbon, and observed with a Zeiss Ultra 55 FEG SEM microscope, operated at 2.0 kV or 10 kV, at a working distance of 2.7 mm or 7.5 mm. Images were acquired in secondary electron mode using a Everhart Thornley or the InLens detectors in backscaterred electron mode. Energy dispersive x-ray spectrometry analyses were performed with an EDS QUANTAX microanalyzer operated with the Esprit, Hypermap software allowing acquisition of X-ray maps and drift correction.

## Results

### The Four *Synechocystis* Genes *sll0923, sll1581, slr1875* and *sll5052* are Dispensable to the Growth of all Corresponding Single and Double Deletion Mutants

Among other *Synechocystis* genes that share sequence homology with EPS production genes from non-cyanobacterial prokaryotes (http://genome.kazusa.or.jp/cyanobase) we decided to study the three chromosomal genes *sll0923*, *sll1581* and *slr1875*, and one plasmidic gene *sll5052*. *slr1875* encodes a presumptive protein homologous to the EPS synthesis ExoD enzyme of *Rhizobium meliloti* [21]; *sll0923* and *sll5052* code for proteins resembling the EPS-assembling enzyme EpsB/GumC/Wzc of *Methylobacillus* [22]; and *sll1581* encodes a presumptive protein homologous to the EPS exporting outer membrane protein GumB/Wza involved in xantham production in *Xanthomonas axonopodis* [23] and biofilm formation in *Xyllela fastidiosa* [24].

**Figure 1:**
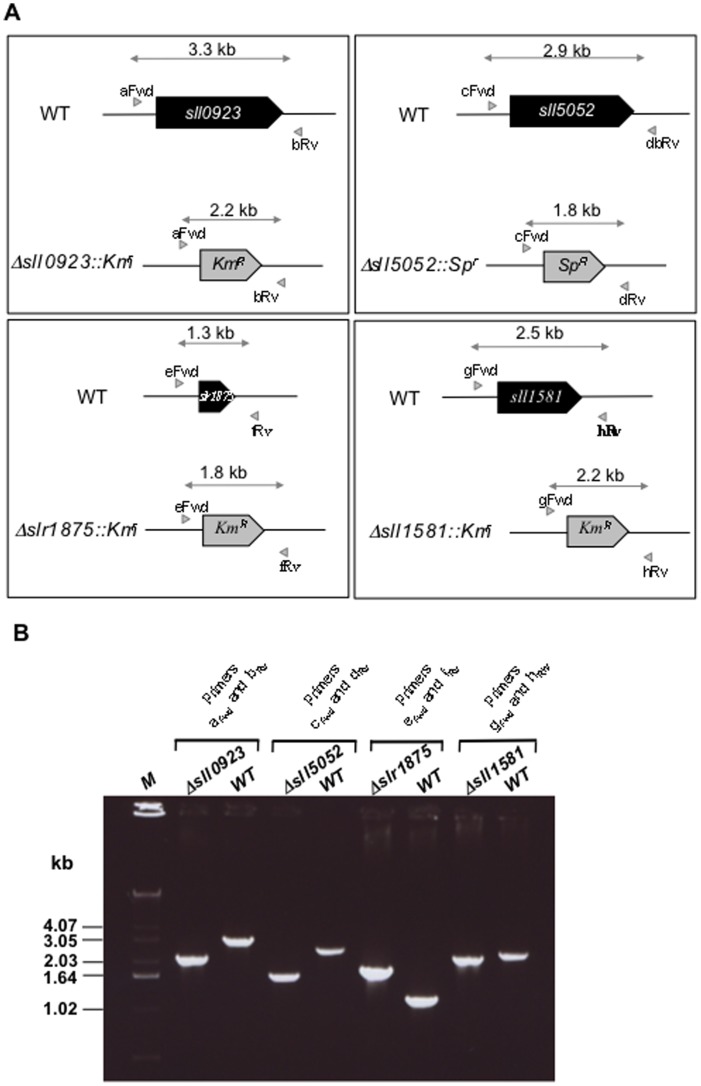
Construction of the single deletion mutants of the genes *sll0923*, *sll1581*, *sll5052* and *slr1875*. (**A**) Schematic representation of the studied chromosome loci in the wild-type (WT) strain and the mutants *Δsll0923*::Km^r^, *Δsll5052*::Sp^r^, *Δslr1875*::Km^r^ and *Δsll1581*::Km^r^ constructed in this study. The studied genes are represented by boxes, which point into the direction of their transcription. The PCR primers used to verify the presence (in the WT strain) and absence (in the deletion mutant) of the studied genes are represented by the small grey triangles. The size of corresponding PCR products, which are represented by the double arrows, are indicated in kilobases (kb). (**B**) Typical UV-light images of the agarose gels showing the PCR products corresponding to the WT and mutant chromosomes. These data show that all deletion mutants harbour no WT copy of the chromosome.

To investigate the four genes *sll0923, sll1581, slr1875* and *sll5052*, we used single and double deletion analysis. Therefore, we replaced the full protein-coding sequence of these genes with a transcription terminator-less marker, Km^r^ or Sm^r/^Sp^r^ (Table 1), for antibiotic selection. The resulting deletion cassettes (Δ*sll0923*::Km^r^, Δ*sll1581*::Km^r^, Δ*slr1875*::Km^r^, Δ*sll5052*::Km^r^, Δ*sll0923*::Sm^r/^Sp^r^, Δ*sll1581*::Sm^r/^Sp^r^, Δ*slr1875*::Sm^r/^Sp^r^ and Δ*sll5052*::Sm^r/^Sp^r^) were independently introduced in *Synechocystis* by transformation. In each case a few transformant clones were selected and analyzed by PCR (Fig. 1 for the Δ*sll0923*::Km^r^, Δ*sll1581*::Km^r^, Δ*slr1875*::Km^r^ and Δ*sll5052*::Sm^r/^Sp^r^ cassettes) and DNA sequencing (data not shown). We verified that the Km^r^ or the Sm^r^/Sp^r^ marker had properly replaced the studied genes in all copies of genome, which is polyploïd [18], [25]. All eight single mutants were found to grow healthy under standard laboratory conditions (Fig. S1), and they retained no wild-type (WT) allele of the studied genes. The complete absence of WT alleles in each mutant was also verified in cultures subsequently grown for about 100 generations in absence of the selective antibiotics.

We also constructed all six possible double mutants *Δsll0923-sll1581*, *Δsll0923-slr1875*, *Δsll0923-sll5052*, *Δslr1875-sll1581*, *Δsll5052*-*sll1581* and *Δsll5052*-*slr1875*, which were selected on the basis of their resistance to all three antibiotics Km, Sm and Sp. The double mutants *Δsll0923-sll1581*; *Δsll0923-slr1875* and *Δsll0923-sll5052* were generated after the introduction of the *Δsll1581*::*Sm^r^/Sp^r^, Δslr1875::Sm^r^/Sp^r^* and *Δsll5052*::*Sm^r^/Sp^r^* cassettes in the *Δsll0923*::*Km^r^* recipient mutant (Fig. S2). The *Δslr1875-sll1581* double mutant was obtained after the introduction of the *Δsll1581*::*Sm^r^/Sp^r^* cassette into the *Δslr1875::Km^r^* recipient mutant (Fig. S3). The *Δsll5052*-*sll1581* and *Δsll5052*-*slr1875* double mutants were generated after the introduction of the *Δsll1581*::Km^r^ and *Δslr1875*::Km^r^ cassettes into *Δsll5052*::*Sm^r^/Sp^r^* recipient mutant (Fig. S4). Like all four single-deletion mutants, all six double mutants grew as healthy as the WT strain (Fig. S1), and lacked WT-type alleles of the studied genes, even after a subsequent cultivation in absence of the selective antibiotics.

These data show that the four *Synechocystis* genes *sll0923, sll1581, slr1875, sll5052* are dispensable to the growth of all corresponding single and double deletion mutants under standard laboratory conditions.

### Influence of the *sll0923*, *sll1581*, *slr1875* and *sll5052* Genes on the Buoyant Density of *Synechocystis*

**Figure 2:**
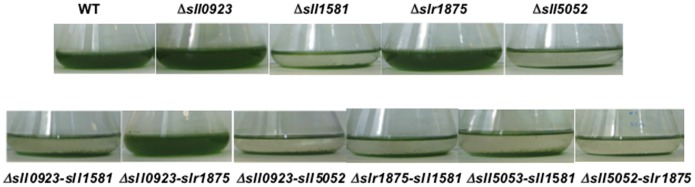
Influence of the *sll0923*, *sll1581*, *sll5052* and *slr1875* genes on the spontaneous cell sedimentation of *Synechocystis*. Typical photographs of cultures of wild-type and mutant strains harbouring either a single or a double deletion of the genes *sll0923*, *sll1581*, *sll5052* and *slr1875*, as indicated. The suspensions were kept static on the bench for 18 days prior to imaging. These experiments were performed at least three times.

Bacterial autoaggregation is the process whereby cells physically interact with each other and settle down to the bottom of culture flask in static liquid cell suspensions. This process is of industrial significance in facilitating the separation of cell biomass from valuable products excreted by the cells in their growth media, such as fatty acids produced by a genetically modified strain of *Synechocystis* [26]. With this in mind, we noticed with great interest that *Synechocystis* spontaneously settles down and forms a “slime” like structure at the bottom of liquid-cultures flasks that are stored under light without agitation. This process is slow and takes more than three weeks in the cases of the WT strain and the three mutants *Δsll0923*, *Δslr1875* and *Δsll0923*-*slr1875*. By contrast, the seven mutants *Δsll1581*, *Δsll5052*, *Δsll5052*-*1581, Δsll0923*-*sll1581, Δsll0923-sll5052, Δslr1875-sll1581* and *Δsll5052*-*slr1875* settled down faster to the bottom of the flask. Their sedimentation was complete in less than 18 days (Fig. 2). Altogether these findings show that the absence of *sll1581* or *sll5052*, but not of *sll0923* and *slr1875*, accelerates the spontaneous cell sedimentation.

### The Three Genes *sll0923*, *sll1581* and *slr1875* Operate in the Production of Exopolysaccharides, While *sll5052* Play a Minor Role

**Figure 3:**
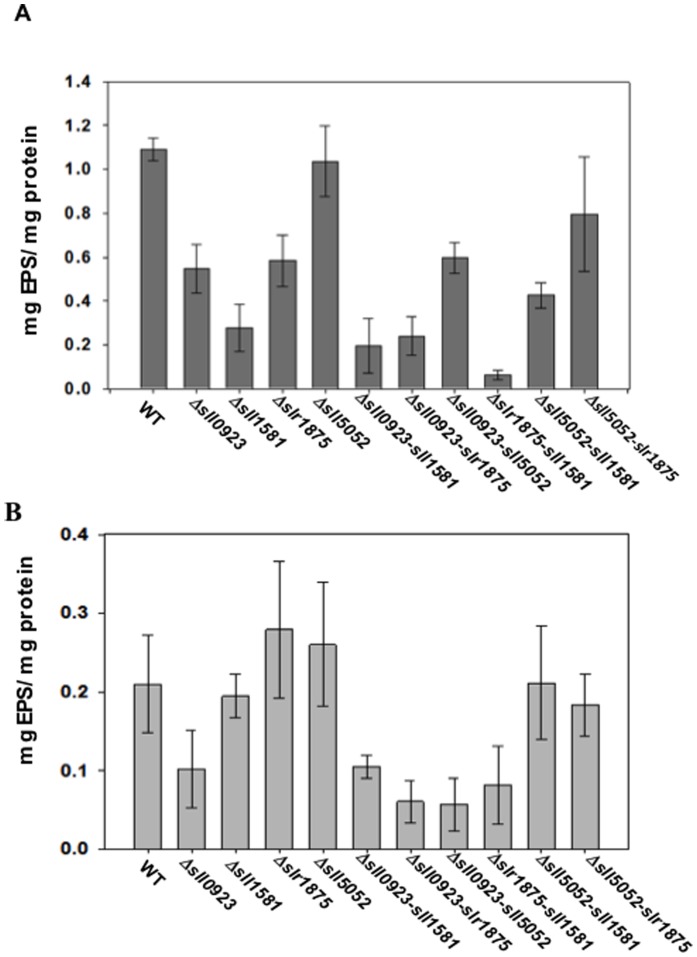
Influence of the *sll0923*, *sll1581*, *sll5052* and *slr1875* genes on exopolysaccharides abundance in *Synechocystis*. EPS amount of the WT and mutant cells harbouring either a single or a double deletion of the genes *sll0923*, *sll1581*, *sll5052* and *slr1875*, as indicated. (**A**) Histogram plots of the amounts of capsular exopolysaccharides (CPS) attached to the cells in each strain. (**B**) Histogram plots of the amounts of exopolysaccharides released (RPS) by each strain in culture medium. All results are expressed as means ± standard deviation of the data obtained after 3 biological repetitions of every assay.

To investigate the influence of the *sll0923, sll1581, slr1875* and *sll5052* genes on EPS production, we measured and compared the abundance of both CPS (capsular polysaccharides attached to cells) and RPS (polysaccharides released in the liquid culture medium) in the WT strain and the 10 studied deletion mutants. As compared to the WT strain (Fig. 3), the quantity of CPS was found to be lower in the three single mutants *Δsll0923* (2-fold decrease), *Δsll1581* (4-fold reduction) and *Δslr1875* (2-fold decrease). Furthermore, the CPS levels were even lower in the three corresponding double mutants *Δsll0923*-*sll1581* (5.5-fold decrease as compared to WT), *Δsll0923*-*Δslr1875* (5-fold reduction), and *Δsll1581*-*slr1875* (11-fold decrease). By contrast, the abundance of CPS was not affected by the deletion of the *sll5052* gene, irrespectively of the strains used for deleting *sll5052*. Indeed (Fig. 3), the amount of CPS were similar in the following pair-wise strain comparisons (i) *Δsll5052* with WT cells; (ii) *Δsll5052-sll0923* with *Δsll0923*; (iii) *Δsll5052-sll1581* with *Δsll1581*; and (iv) *Δsll5052-slr1875* with *Δslr1875*. Together, these findings show that the *sll0923, sll1581, slr1875* genes operate in the quantitative production of CPS, unlike *sll5052*.

The abundance of the RPS polysaccharides was decreased about 2 fold in the *Δsll0923* mutant, whereas it was unaffected in *Δsll1581*, and even slightly increased in the *Δslr1875* and *Δsll5052* mutants, as compared to the WT strain (Fig. 3). These results suggest that the three genes *sll1581*, *slr1875* and *sll5052* are dispensable to RPS production. However, this interpretation is challenged by the findings that most of the corresponding double deletion mutants exhibit less RPS than the WT strain and their parental single deletion mutants. For instance, the RPS abundance in the double mutant *Δsll1581*-*slr1875* is at least two-fold lower than that in the WT strain and the two single mutants *Δsll1581* and *Δslr1875*. Similarly, the RPS abundance of the mutant *Δsll0923* is decreased upon the secondary deletion of any of the *sll1581*, *slr1875* and *sll5052* genes.

Collectively these findings show that the *sll0923* gene operates in the production of both CPS and RPS, whereas *sll1581* and *slr1875* operate in the production of mainly CPS and moderately RPS, and *sll5052* is possibly involved in the production of RPS, likely not of CPS.

### The Abundance of *Synechocystis* EPS Influences the Zeta Potential of the Cell

**Figure 4:**
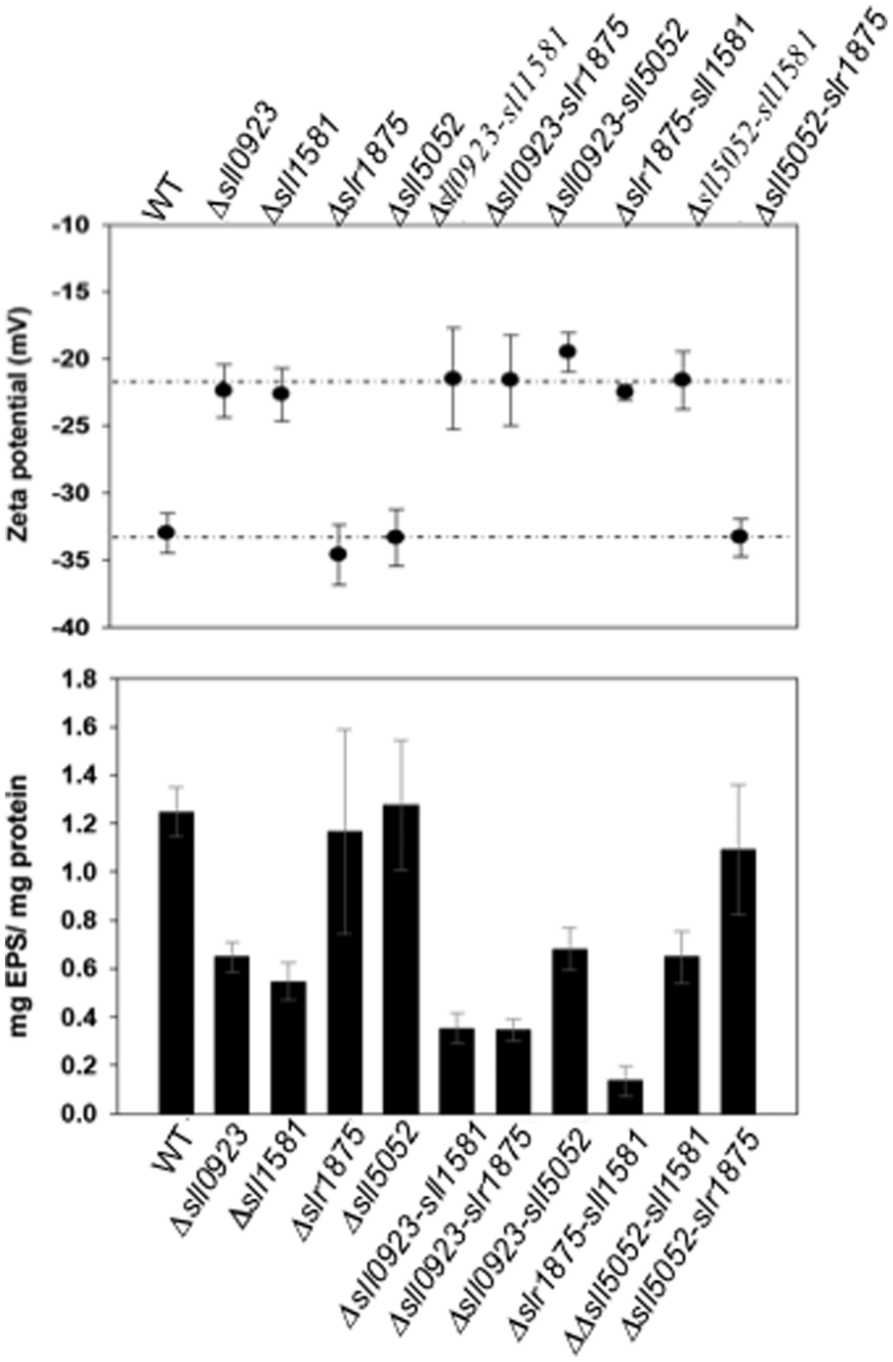
The abundance of total EPS influences the zeta potential of *Synechocystis*. (**A**) Zeta values for the WT and mutant cells harbouring either a single or a double deletion of the genes *sll0923*, *sll5052*, *slr1875* and *sll1581*, as indicated. (**B**) Histogram plots of the total amounts of EPS (CPS+RPS) of each strain. All results are expressed as means ± standard deviation of the data obtained after three biological repetitions of every assay.

Bacterial surface charge, which influences cell interactions with the medium and the other cells (cell-to-cell aggregation and biofilm formation), can be assessed by measuring the zeta potential that can be deduced from electrophoretic mobility measurements. Thus, we determined and compared the zeta potential of the WT strain (≈ −33 mV, as we shown [20]) with the values observed for the ten presently-studied mutants in order to assess the influence of EPS on the cell surface of *Synechocystis* (Fig. 4). The single mutants *Δslr1875* and *Δsll5052* and the corresponding double mutant *Δsll5052*-slr*1875* displayed zeta potentials (−34.6 mV, −33.3 mV and −33.3 mV, respectively) similar to WT cells (Fig. 4). By contrast, less-negative values were observed for the other mutants *Δsll0923*, *Δsll1581*, *Δsll0923*-*sll1581*, *Δsll0923-sll5052, Δslr1875-sll1581* and *Δsll5052-sll1581* (between −20 mV and −25 mV; Fig. 4 panel A), which produce only about half or less of the WT amount of EPS (Fig. 4 panel B). Collectively, these finding indicate that the zeta potential is correlated with the total amount of EPS and the resulting density of ionic surface charges produced by the cells (Fig. 4, compare panels A and B). In turn, our data suggest that zeta potential assays should be used in the future for facile screening of EPS-depleted mutants.

### The Strong Reduction in EPS Content Caused by the Double Deletion of *slr1875 and sll1581* Decreases the Tolerance of *Synechocystis* to Salt and Heavy Metals Stresses

**Figure 5:**
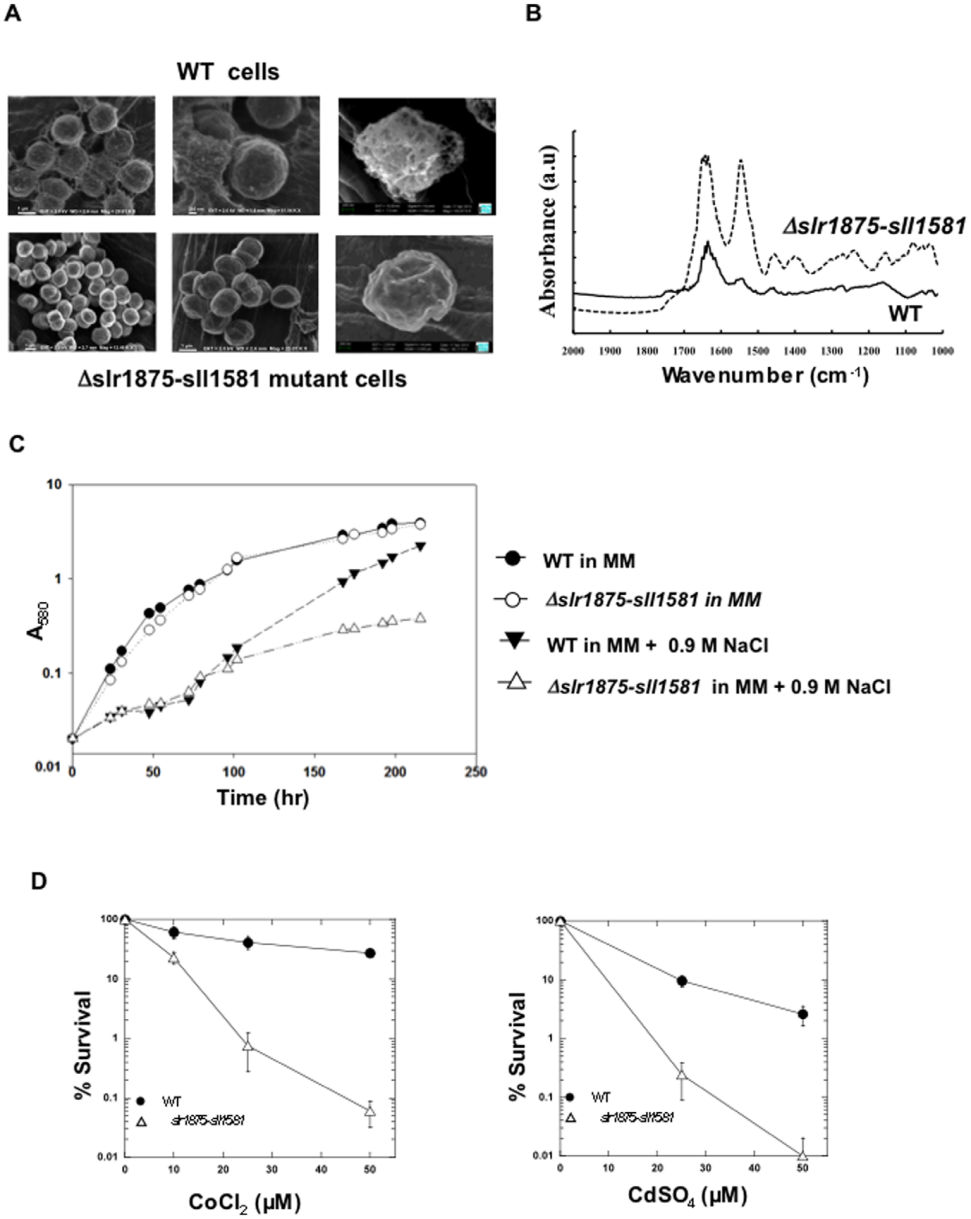
Influence of *Synechocystis* exopolysaccharides on cell shape and tolerance to salt and heavy metal stresses. (**A**) Typical SEM (scanning electron microscopy) images of WT cells and *Δslr1875-sll1581* double mutant cells. (**B**) Typical FTIR absorption spectra of WT cells (solid line) and EPS-depleted *Δslr1875-sll1581* cells (dashed lines). (**C**) Typical growth of the WT strain (dark symbols) and EPS-depleted *Δslr1875-sll1581* double mutant (open symbols) incubated for the indicated durations in standard liquid mineral medium (MM) without or with NaCl 0.9 M. (**D**) Typical survival of the WT strain (dark circles) and the *Δslr1875-sll1581* double mutant (open triangles) challenged with CoCl_2_ and CdSO_4_. All experiments were performed at least three times.

To study the influence of EPS in the protection against the frequently encountered salt and heavy metal stresses we used the *Δslr1875-sll1581* double mutant, which possesses the lowest EPS content (Fig. 3 and Fig. 4). First, we confirmed the difference in EPS content between WT cells and *Δslr1875-sll1581* cells through (i) SEM microscopy, which showed that these mutant cells lack the copious EPS mantle that wraps the WT cells (Fig. 5 panel A) and (ii) FTIR absorption analysis, which confirmed the difference in cell surface between EPS-replete WT cells and EPS-depleted *Δslr1875-sll1581* cells (Fig. 5 panel B). Then, we showed that *Δslr1875-sll1581* cells, which grow as healthy as WT cells in absence of stress, displayed an increased susceptibility to NaCl (Fig. 5 panel C), cadmium (CdSO_4_) and cobalt (CoCl_2_) (Fig. 5 panel D).

### Exopolysaccharides Sorb Iron and Protect Cells from Iron Starvation

**Figure 6:**
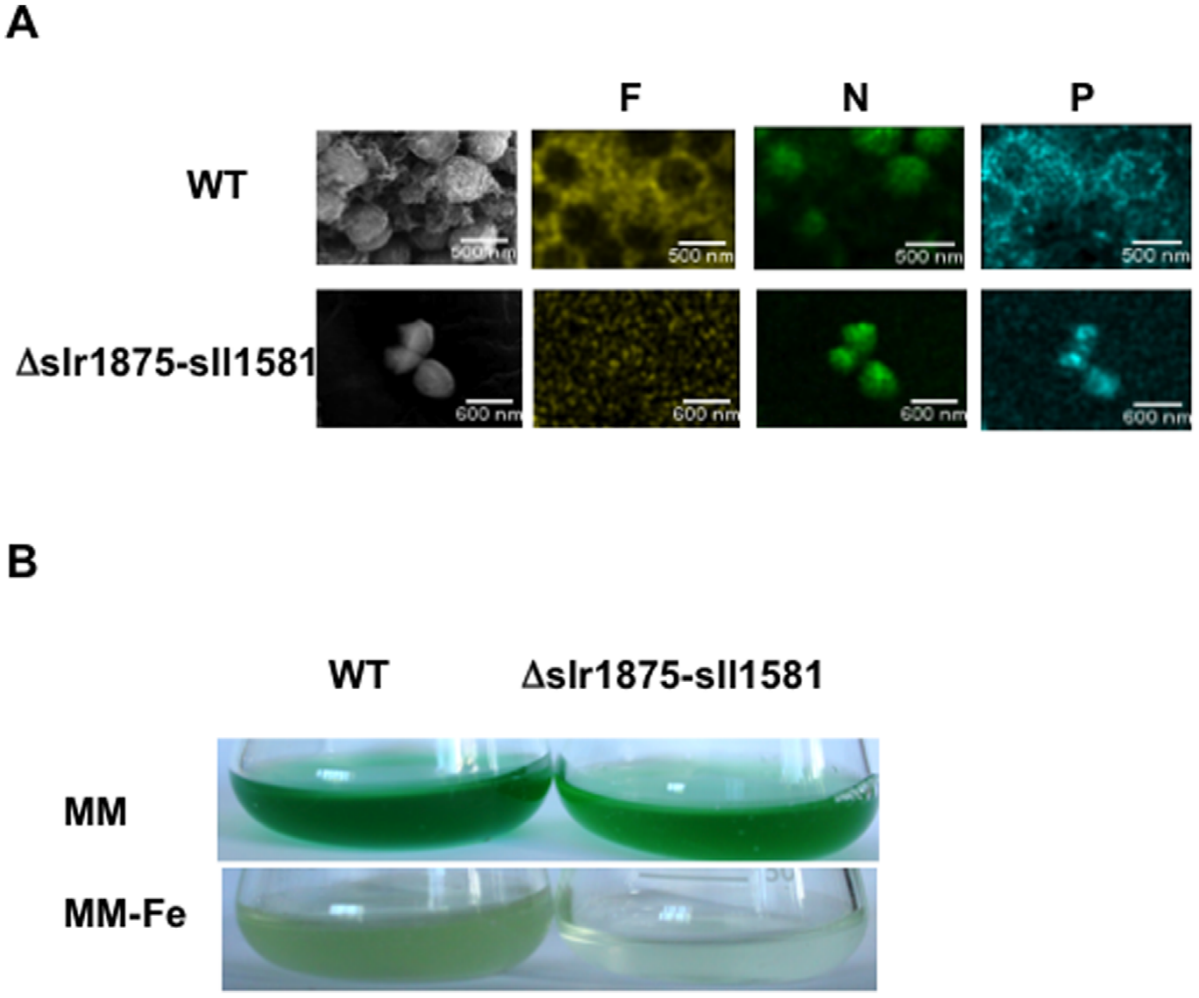
SEM-EDXS of WT and EPS-depleted *Δ* *slr1875-sll1581* cells. (**A**) SEM-EDXS images and chemical maps (scale bars, 300 nm) of WT and *Δ*slr1875-sll1581 cells grown on liquid MM medium. (**B**) Photographs of typical cultures of WT and EPS-depleted *Δ*slr1875-sll1581 cells incubated for eight days in MM (containing 17 µM iron) or Fe-less MM, as indicated.

Our evidence that EPS play a direct role in the protection against both Cd and Co (Fig. 5), which disturb iron (Fe) homeostasis [27], [28], which is especially important in cyanobacteria [29], prompted us to test whether EPS might operate in the sorption of Fe. Therefore, we analyzed WT and EPS-depleted mutant *Δslr1875-sll1581* cells by SEM (in secondary and backscattered electron modes) and energy-dispersive x-ray spectroscopy (EDXS) analyses of the same cells. We observed that similarly to EPS, Fe is highly abundant in the vicinity of WT, not *Δslr1875-sll1581*, cells (Fig. 6A), like EPS. This co-localization indicates that Fe is trapped by the abundant EPS surrounding WT cells. In comparison, nitrogen (N), which is mostly associated with intracellular molecules (amino-acids, proteins and nucleic acids), displayed a similar intracellular localization in both WT and mutant cells. In addition, phosphorus (P) was observed both inside the cells and as EPS-chelated materials (Fig. 6A). Then, we showed that the WT strain can grow for some time in absence of iron, unlike strain the EPS-depleted *Δslr1875-sll1581* mutant (Fig. 6B). Altogether, these findings show that EPS play a role in Fe-sorption, which protects *Synechocystis* against iron starvation.

## Discussion

Little is known in cyanobacteria about the production of exopolysaccharides (EPS), in spite of their proposed involvement in the protection against environmental stresses [7]. Even in the best-characterized unicellular cyanobacterium, *Synechocystis* PCC6803 (*Synechocystis*), only a few genes, *sll1722* to *sll1726*, have been identified as operating in EPS production [10]. Furthermore, there is no strong evidence in the literature that EPS play a direct role in the protection of cyanobacteria against salt and metal stresses, which they frequently encounter in nature. This scarcity of information prompted us to study four *Synechocystis* genes, *sll0923*, *sll1581*, *slr1875* and *sll5052* that share sequence homology with EPS production genes from other bacteria (http://genome.kazusa.or.jp/cyanobase/). These four *Synechocystis* genes appeared to be dispensable to cell growth under standard laboratory conditions, of all corresponding single and double deletion mutants. Both *sll1581* and *sll5052* play a role in the buoyant density of *Synechocystis*, unlike both *sll0923* and *slr1875*. Indeed, all single or double deletion mutants of *sll1581* and *sll5052* displayed an accelerated sedimentation of the cells down to the bottom of the flask in static suspensions (Fig. 2). Such fast sedimenting mutants might be useful cell factories for the future production of biotechnologically interesting products, in the sense that they should facilitate the otherwise expensive harvest of the producer biomass and/or the separation of products excreted in the growth medium. We also show that the *sll0923*, *sll1581* and *slr1875* genes unambiguously operate in the quantitative production of both CPS (capsular) and RPS (released) EPS, unlike *sll5052* (Fig. 3). Both *sll1581* and *slr1875* are more important than *sll0923* for CPS production, whereas the contrary was true for RPS production. Our findings that EPS-depleted *Synechocystis* mutants can form biofilms, even faster than the WT strain (Fig. 2), are consistent with previous EPS studies in many other bacteria including *E. coli, P. putida, S. haemolyticus, S. pneumoniae, etc* [4], [30]. These investigations identified several EPS that inhibit biofilm formation in acting as surfactants, rather than bactericidal agents. As these antibiofilm EPS destabilize biofilm matrix without affecting bacterial fitness, unlike antibiotics, they should be less prone than antibiotics to develop resistance. Hence, these antibiofilm EPS should have interesting potentials in industry and medicine [30].

We also studied the influence of EPS on the cell surface charge of *Synechocystis*, estimated as the zeta potential value. We found that the mutants, *Δsll0923*, *Δsll1581*, *Δsll0923*-*sll1581*, *Δsll0923-sll5052, Δslr1875-sll1581* and *Δsll5052-sll1581*, which produce about half of the WT amount of EPS, displayed higher zeta potentials than the WT strain (Fig. 4). These data indicate that the zeta potential of cells is correlated with the total amount of EPS and the associated density of ionic surface charges. Thus, we suggest that zeta potential assays should be systematically used in the future for facile screening of EPS production mutants. Then, we thoroughly analyzed the most EPS-depleted mutant, *Δslr1875-sll1581* (17 -fold lower EPS abundance than the WT strain) and the WT strain with various microscopy techniques. We show that *Δslr1875-sll1581* cells lack the copious EPS mantle that surrounds WT cells (Fig. 5) and massively stores Fe in their vicinity (Fig. 6). Consistently, we found that the EPS protect cells against the starvation of Fe (Fig. 6), which is crucial to cyanobacteria, and often limited in their natural environments [29]. Hence we propose that similarly to siderophores, which are not produced by *Synechocystis* [29], EPS operate in Fe homeostasis in sorbing Fe, which can be subsequently released and taken up by the cells when required. Finally, we show (Fig. 5) that the EPS protect cells from the toxicity of NaCl, CdSO_4_) and CoCl_2_, which are frequently encountered by cyanobacteria in nature [9], [31]. Our results support the notion that EPS, in surrounding cells, behave as a physico-chemical barrier preventing direct contacts between cells and toxics. Furthermore, our data are consistent with the previous findings that the toxicity of both Cd and Co disturb Fe homeostasis, and can be decreased by increasing Fe availability [27], [28]. Collectively, our results support the proposed utilization of EPS-producing cyanobacteria as chelating agents for biosorption processes aiming at removing toxic heavy metals from contaminated waters [7]. In addition, we believe that our mutants defective in EPS formation will be of help to better understand the role of cyanobacterial EPS in autoaggregation, protection against environmental stresses, and the biomineralization of the carbon dioxide gas by calcium and/or magnesium carbonates precipitation. This latter objective has both a basic research interest and an interesting biotechnological potential for carbon capture and storage [8]. We also suggest to investigate the biotechnological potentials offered by fast sedimenting EPS mutants, such as those presented here, for the characterization of potential surfactants, and to serve as cell factories that can be easily harvested and/or separated from products excreted in the growth media.

## Supporting Information

Figure S1 **Typical growth curves of the WT strain and the ten EPS-depleted mutants.**
(TIF)

Figure S2 **PCR analysis of the double deletion mutants** ***Δsll0923-sll1581*** **,** ***Δsll0923-slr1875*** **and** ***Δsll0923-sll5052.*** These double mutants were generated after the introduction of the *Δsll1581::Sm^r^/Sp* ^r^, *Δslr1875::Sm^r^/Sp^r^* and *Δsll5052::Sm^r^/Sp^r^* cassettes in the *Δsll0923::Km^r^* recipient mutant. Left panels : Schematic representation of the studied chromosome loci in the wild-type (WT) strain and the corresponding deletion mutants. The studied genes are represented by boxes pointing into the direction of their transcription. The size of the PCR products (double arrows) generated by the primers (small triangles) are indicated in kb. Right panels Typical UV-light images of the agarose gels showing the PCR products corresponding to the WT and mutant chromosomes. These data show that all studied clones of the deletion mutants harbour no WT copy of the chromosome. M_1_ and M_50_ respectively indicate the 1 kb DNA ladder and 50 bp DNA ladder (Invitrogen).
(TIF)

Figure S3 **Construction of the double deletion mutant** ***Δslr1875-sll1581.*** The *Δslr1875-sll1581* double mutant was obtained after introduction of *Δsll1581*::*Sm^r^/Sp^r^* cassette into the *Δslr1875::Km^r^* recipient mutant. Left panel : Schematic representation of the studied chromosome loci in the wild-type (WT) strain and the corresponding deletion mutant. The studied genes are represented by boxes pointing into the direction of their transcription. The size of the PCR products (double arrows) generated by the primers (small triangles) are indicated in kb. Right panels Typical UV-light images of the agarose gels showing the PCR products corresponding to the WT and mutant chromosomes. These data show that all studied clones of the deletion mutant harbour no WT copy of the chromosome. M_1_ and M_50_ respectively indicate the 1 kb DNA ladder and 50 bp DNA ladder (Invitrogen).
(TIF)

Figure S4 **PCR analysis of the double deletion mutant** *Δ* ***sll5052*** **-** ***sll1581*** **and** *Δ* ***sll5052*** **-** ***slr1875.*** These double mutants were generated after the introduction of the *Δsll1581*::Km^r^ and *Δslr1875*::Km^r^ cassettes into the *Δsll5052*::*Sm^r^/Sp^r^* recipient mutant. Left panels : Schematic representation of the studied chromosome loci in the wild-type (WT) strain and the corresponding deletion mutants. The studied genes are represented by boxes pointing into the direction of their transcription. The size of the PCR products (double arrows) generated by the primers (small triangles) are indicated in kb. Right panels Typical UV-light images of the agarose gels showing the PCR products corresponding to the WT and mutant chromosomes. These data show that all studied clones of the deletion mutants harbour no WT copy of the chromosome. M_1_ and M_50_ respectively indicate the 1 kb DNA ladder and 50 bp DNA ladder (Invitrogen).
(TIF)

Table S1 **Sequence of the PCR primers used in this study.**
(PDF)
