# Supplementary figures and images for: Multidisciplinary Evidences that Synechocystis PCC6803 Exopolysaccharides Operate in Cell Sedimentation and Protection against Salt and Metal Stresses
Source: PLoS One. 2013 Feb 6;8(2):e55564. doi: 10.1371/journal.pone.0055564 (PMC3566033; doi:10.1371/journal.pone.0055564)

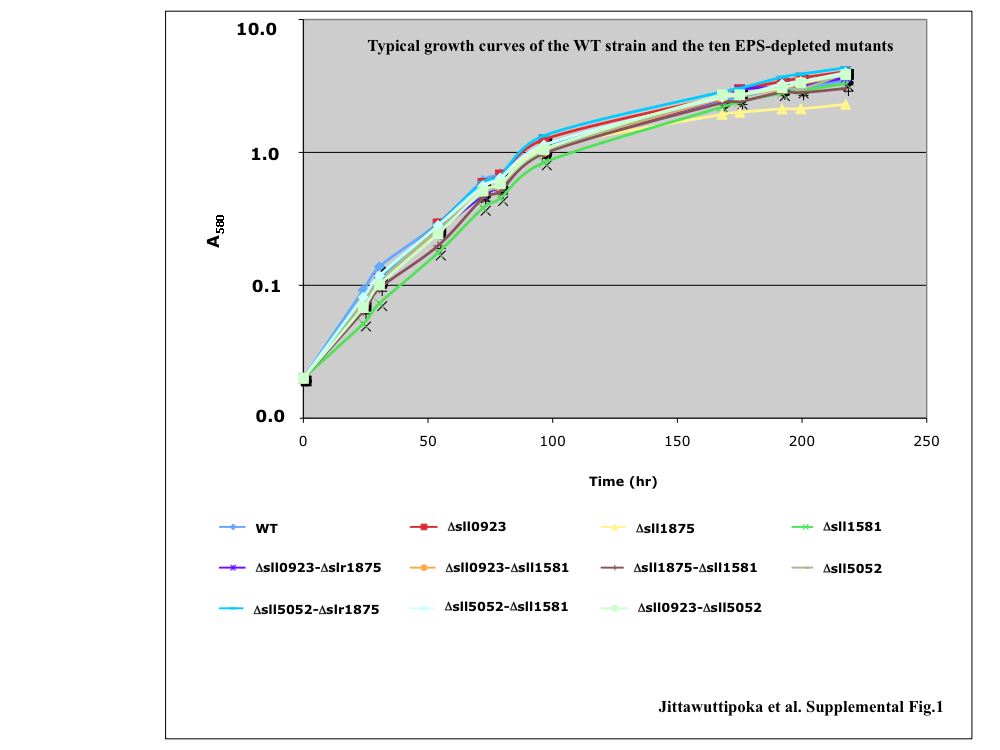

Supplement: Figure S1 — Typical growth curves of the WT strain and the ten EPS-depleted mutants. (TIF) [file pone.0055564.s001.tif]

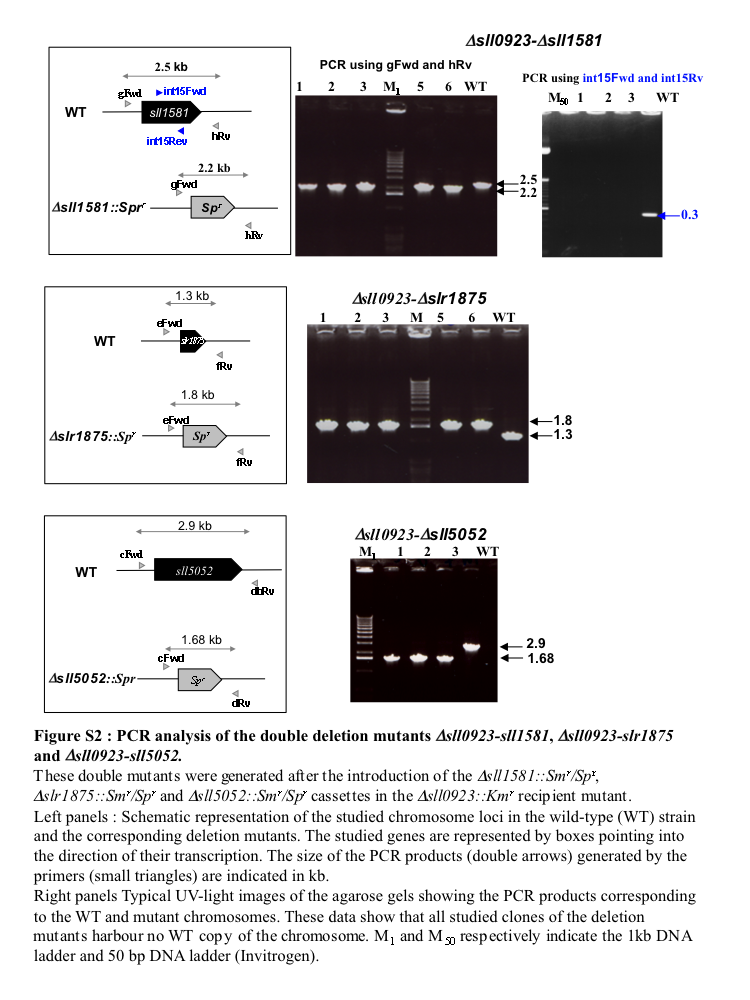

Supplement: Figure S2 — PCR analysis of the double deletion mutants Δsll0923-sll1581 , Δsll0923-slr1875 and Δsll0923-sll5052. These double mutants were generated after the introduction of the Δsll1581::Smr/Sp r, Δslr1875::Smr/Spr and Δsll5052::Smr/Spr cassettes in the Δsll0923::Kmr recipient mutant. Left panels : Schematic representation of the studied chromosome loci in the wild-type (WT) strain and the corresponding deletion mutants. The studied genes are represented by boxes pointing into the direction of their transcription. The size of the PCR products (double arrows) generated by the primers (small triangles) are indicated in kb. Right panels Typical UV-light images of the agarose gels showing the PCR products corresponding to the WT and mutant chromosomes. These data show that all studied clones of the deletion mutants harbour no WT copy of the chromosome. M1 and M50 respectively indicate the 1 kb DNA ladder and 50 bp DNA ladder (Invitrogen). (TIF) [file pone.0055564.s002.tif]

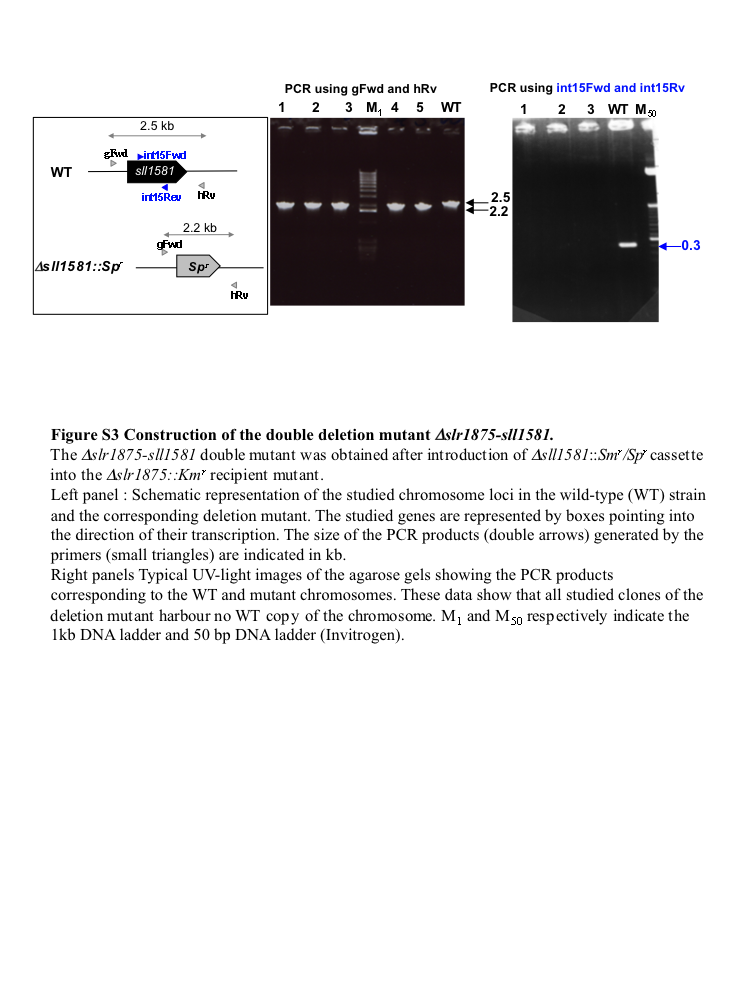

Supplement: Figure S3 — Construction of the double deletion mutant Δslr1875-sll1581. The Δslr1875-sll1581 double mutant was obtained after introduction of Δsll1581::Smr/Spr cassette into the Δslr1875::Kmr recipient mutant. Left panel : Schematic representation of the studied chromosome loci in the wild-type (WT) strain and the corresponding deletion mutant. The studied genes are represented by boxes pointing into the direction of their transcription. The size of the PCR products (double arrows) generated by the primers (small triangles) are indicated in kb. Right panels Typical UV-light images of the agarose gels showing the PCR products corresponding to the WT and mutant chromosomes. These data show that all studied clones of the deletion mutant harbour no WT copy of the chromosome. M1 and M50 respectively indicate the 1 kb DNA ladder and 50 bp DNA ladder (Invitrogen). (TIF) [file pone.0055564.s003.tif]

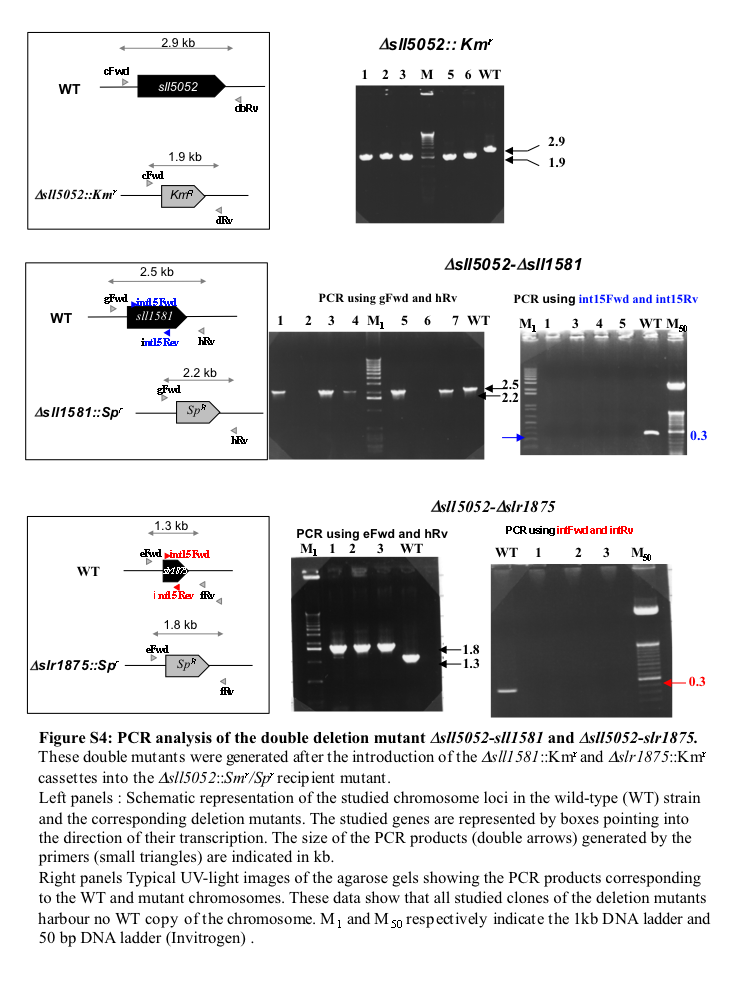

Supplement: Figure S4 — PCR analysis of the double deletion mutant Δ sll5052 - sll1581 and Δ sll5052 - slr1875. These double mutants were generated after the introduction of the Δsll1581::Kmr and Δslr1875::Kmr cassettes into the Δsll5052::Smr/Spr recipient mutant. Left panels : Schematic representation of the studied chromosome loci in the wild-type (WT) strain and the corresponding deletion mutants. The studied genes are represented by boxes pointing into the direction of their transcription. The size of the PCR products (double arrows) generated by the primers (small triangles) are indicated in kb. Right panels Typical UV-light images of the agarose gels showing the PCR products corresponding to the WT and mutant chromosomes. These data show that all studied clones of the deletion mutants harbour no WT copy of the chromosome. M1 and M50 respectively indicate the 1 kb DNA ladder and 50 bp DNA ladder (Invitrogen). (TIF) [file pone.0055564.s004.tif]
