# Supplementary material for: Multidisciplinary Evidences that Synechocystis PCC6803 Exopolysaccharides Operate in Cell Sedimentation and Protection against Salt and Metal Stresses
Source: PLoS One. 2013 Feb 6;8(2):e55564. doi: 10.1371/journal.pone.0055564 (PMC3566033; doi:10.1371/journal.pone.0055564)
Supplement: Table S1 — Sequence of the PCR primers used in this study. (PDF) [file pone.0055564.s005.pdf]

Supplemental Table 1: Sequence of the PCR primers used in this study

**Inactivation of *sll0923***		
sll0923 Up Fwd	CGGTTACAGACAAATCTAATTTATTGAC	Forward primer for amplification of *sll0923* upstream region
sll0923 Up Rv	AACCCCGGGGCAATTCTGGGTAACTGTAGG	Reverse primer for amplification of *sll0923* upstream region with a *Sma*I site
sll0923 Down Fwd	AATTGCCCCGGGGTTATGGCTACTACTATGGTC	Forward primer for amplification of *sll0923* downstream region with a *Sma*I site
sll0923 Down Rv	GACAGGAATGGATGTCCAGATCCGGGC	Reverse primer for amplification of *sll0923* downstream region
sll0923 Fwd	CATTGAGCTAGATATTCTC	Forward primer for verification of *sll0923* segregation
sll0923 Rv	CCAAAAGTCCCAGCATGA	Reverse primer for verification of *sll0923* segregation
**Inactivation of *sll1581***		
sll1581 Up Fwd	TTAATGACTCGGAGGTTATTTTAGCGC	Forward primer for amplification of *sll1581* upstream region
sll1581 Up Rv	AACCCCGGGGAGTTTAGAAGGGAAGGATTG	Reverse primer for amplification of *sll1581* upstream region with a *Sma*I site
sll1581 Down Fwd	AAACTCCCCGGGGTTTAACTCAAGCTACCG	Forward primer for amplification of *sll1581* downstream region with a *Sma*I site
sll1581 Down Rv	GTTGCTTGCAGGCATAATTTTTGGAGC	Reverse primer for amplification of *sll1581* downstream region
sll1581 Fwd	GGTCACTGCCATTCTGAC	Forward primer for verification of *sll1581* segregation
sll1581 Rv	CTGCTTATAAGCTGACATTC	Reverse primer for verification of *sll1581* segregation
**Inactivation of *slr1875***		
slr1875 Up Fwd	TGGATGCGGCGGGCTTAGGACGATTGG	Forward primer for amplification of *slr1875* upstream region
slr1875 Up Rv	GTAATACCCGGGACAAACGGGCCATATGGATAG	Reverse primer for amplification of *slrl1875* upstream region with a *Sma*I site
slr1875 Down Fwd	TGTCCCGGGTATTACCATCATTGACATTC	Forward primer for amplification of *slrl1875* downstream region with a *Sma*I site
slr1875 Down Rv	GTCCACTTCGACGGCTTAGCCATTGTTTG	Reverse primer for amplification of *slrl18755* downstream region
sll1875 Fwd	GCAATTCAATTGATGGCTG	Forward primer for verification of *slrl1875* segregation
sll1875 Rv	GCCATCATCCTCCTGGA	Reverse primer for verification of *slrl1875* segregation
**Inactivation of *sll5052***		
sll5052 Up Fwd	ACGAAAGACTCTCTGGACAGATAAAAG	Forward primer for amplification of *sll5052* upstream region
sll5052 Up Rv	TTTCCCGGGTAATCGCCACCATCTGAGGG	Reverse primer for amplification of *sll5052* upstream region with a *Sma*I site
sll5052 Down Fwd	CGATTACCCGGGAAAAGCGACAATGATAAACC	Forward primer for amplification of *sll5052* downstream region with a *Sma*I site
sll5052 Down Rv	TTCGTGCTTGACCGGTGTTTAACTTAAG	Reverse primer for amplification of *sll5052* downstream region
sll5052 Fwd	GCCAAATTCAAGATCCCAC	Forward primer for verification of *sll5052* segregation
sll5052 Rv	CCAACTGTACATGGGGAC	Reverse primer for verification of *sll5052* segregation

Amplification of the Km^{r} cassette

KmHinCFW	GGCGCTGAG**GTCGAC**CTCGTGAAGAAG	Forward primer for amplification of Km^{r} to be cloned as a *Hinc*II fragment
KmHinCRV	ACCTGCAGGGG**GTCGAC**GGAAAGCCAC	Reverse primer for amplification of Km^{r} to be cloned as a *Hinc*II fragment

Amplification of the Smr/Spr Cassette

SmR Fwd EcoRV pFC1	GGATGAAGGCACGATATCAGTGGACATAAGCC	Forward primer for Sp^{r}/Sm^{r} with an *Eco*RV site
SmR Rv EcoRV pFC1	CCGCGAAGCGGCGATATCTTGAACGAATTGTTAG	Reverse primer for Sp^{r}/Sm^{r} with *Eco*RV site
